# Galactan mobilization during carbon starvation compromises plant cell wall‐mediated resistance to fungal infection

**DOI:** 10.1111/tpj.70438

**Published:** 2025-08-21

**Authors:** Louisa Reckleben, Richard N. Morton, Kristina S. Munzert‐Eberlein, Sabine Thiel, Carsten Rautengarten, Viviana Tan, Klara L. Harres, Lena Zanoni, Berit Ebert, Timo Engelsdorf

**Affiliations:** ^1^ Molecular Plant Physiology, Department of Biology Philipps‐Universität Marburg 35043 Marburg Germany; ^2^ Faculty of Biology and Biotechnology Ruhr University Bochum 44801 Bochum Germany

**Keywords:** cell wall, pectin, galactose, sugar salvage, starch, phytopathogenic fungi, pathogen resistance, pathogen defense, *Arabidopsis thaliana*, *Colletotrichum higginsianum*

## Abstract

Polysaccharides are the main components present in plant cell walls. They form a network that is dynamically modified during growth and upon both abiotic and biotic stress. We investigated how the cell wall of Arabidopsis rosettes is remodeled during periods of dark‐induced starvation in the wild type and in plastidic *phosphoglucomutase* (*pgm*) mutants, which suffer from periodic starvation due to starch deficiency. Time‐course analysis demonstrated that up to one fifth of the galactose present in leaf cell walls is reversibly released upon starvation, while other cell wall monosaccharides were less affected. An investigation of *β‐galactosidase* (*BGAL*) expression and the analysis of *bgal* mutants indicated that *BGAL1* and *BGAL4* contribute to the release of cell wall galactose upon starvation. Increased transcript abundance of *UDP‐glucose 4‐epi* (*UGE*) *1* and *3* under starvation proposed an increased flux through the galactose salvage pathway; however, an analysis of the UDP‐galactose pool in mutant plants indicated redundancy with other UGEs. Simultaneously to galactan degradation, *Galactan synthase* (*GALS1*) expression was reduced, attenuating the synthesis of new galactan chains. We show that overexpression of GALS1 prevents depletion of the recyclable cell wall galactose pool and is sufficient to rescue impaired penetration resistance to the hemibiotrophic fungal pathogen *Colletotrichum higginsianum* upon dark‐induced and periodic starvation. Our data suggest that pectic galactan in the plant cell wall serves as a sugar resource during starvation conditions. However, galactose release from the wall leads to impaired penetration resistance against a fungal pathogen, causing a trade‐off between sugar supply for plant metabolism and preformed defense.

## Graphical abstract

Polysaccharides present in plant cell walls must be dynamically modified in response to external stimuli, but it is not well understood how cell wall remodeling is adjusted in response to metabolic cues and how this affects defense against pathogens. Here, we report that galactose is reversibly released from cell walls upon carbon starvation, which critically affects penetration resistance to a fungal pathogen.

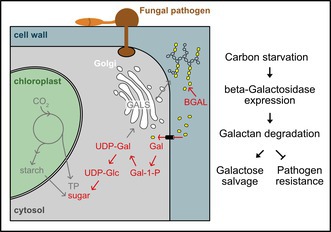

## INTRODUCTION

Cell walls provide support to plant cells and delimit them against neighboring cells and their environment. Polysaccharides represent the main components of the wall and form a network of cellulose, hemicelluloses, and pectins. This network is dynamically remodeled during growth and development, as well as in response to stress (Delmer et al., 2024). When carbon supply from photosynthesis is limited, alterations in cell wall composition occur that might be linked to carbon provision from cell wall polymers. Dark‐induced sugar starvation in detached *Arabidopsis thaliana* (hereafter: Arabidopsis) leaves leads to the induction of glycoside hydrolases and to reduced amounts of pectin and hemicellulose after 2 days of starvation (Lee et al., 2007). Carbon depletion of intact Arabidopsis plants caused by an extended dark phase leads to distinct transcriptional changes aimed at adapting metabolism to the low carbon availability (Usadel et al., 2008). Responses are similar in starch‐deficient plastidic *phosphoglucomutase* (*pgm*) mutants at the end of the night, when they suffer carbon limitation after soluble sugars are depleted (Caspar et al., 1985; Engelsdorf et al., 2013; Gibon et al., 2004; Usadel et al., 2008). This periodic nocturnal carbon limitation in *pgm* causes pronounced alterations in leaf cell wall composition. Cell wall glycan profiling with monoclonal antibodies indicated a reduced presence of epitopes from pectic rhamnogalacturonan‐I (RG‐I) side chains, and cell wall monosaccharide analysis revealed reduced relative amounts of arabinose and galactose in the *pgm* mutant (Engelsdorf et al., 2017).

RG‐I consists of a backbone of alternating galacturonic acid and rhamnose units decorated with arabinan, galactan, and arabinogalactan side chains and can constitute up to one‐third of all pectins in leaf primary cell walls (Mohnen, 2008). The remaining pectin is comprised of homogalacturonan (up to two‐thirds), consisting entirely of galacturonic acid, and of substituted homogalacturonan, such as RG‐II and xylogalacturonan (Delmer et al., 2024). Nucleotide sugars, primarily made in the cytosol, serve as substrates for polysaccharide formation (Bar‐Peled & O'Neill, 2011). UDP‐D‐glucose represents the central intermediate in the nucleotide sugar interconversion pathway and can be directly interconverted to UDP‐D‐glucuronic acid, UDP‐L‐rhamnose, and UDP‐D‐galactose (Bar‐Peled & O'Neill, 2011). In Arabidopsis, interconversion of UDP‐D‐glucose and UDP‐D‐galactose is catalyzed by a family of five UDP‐glucose 4‐epimerases (UGEs), with the expression of *UGE1* and *UGE3* being co‐regulated with carbohydrate catabolism and that of *UGE2*, *UGE4*, and *UGE5* with carbohydrate biosynthesis (Barber et al., 2006). UDP‐rhamnose/UDP‐galactose transporters (URGTs) translocate the substrates for galactan biosynthesis into the Golgi lumen (Rautengarten et al., 2014). After import into the Golgi, nucleotide sugars are used as substrates by glycosyltransferases to assemble cell wall polysaccharides. RG‐I:Rhamnosyltransferase (RRT) and RG‐I:Galacturonosyltransferase (RGGAT) build the RG‐I backbone, while UDP‐D‐galactose serves as substrate for the synthesis of β‐1, 4‐galactan catalyzed by the galactosyltransferases Galactan synthase 1 (GALS1), GALS2, and GALS3 (Amos et al., 2022; Ebert et al., 2018; Liwanag et al., 2012; Takenaka et al., 2018). GALS1 is furthermore able to regulate the length of galactan chains by terminally adding arabino*pyranose* (Ara*p*) (Laursen et al., 2018). The nucleotide sugar UDP‐L‐Ara*p* is formed in the Golgi via sequential conversion of UDP‐D‐glucose to UDP‐D‐glucuronic acid and UDP‐D‐xylose and the subsequent activity of UDP‐xylose 4‐epimerases (UXEs) (Mariette et al., 2021). In Arabidopsis, Murus4 (MUR4) represents the main Golgi‐localized UXE isoform, whereas UGE1 and UGE3 exhibit additional cytosolic UXE activity (Burget et al., 2003; Kotake et al., 2009; Umezawa et al., 2024). In the cytosol, UDP‐L‐Ara*p* is converted to UDP‐L‐arabino*furanose* (UDP‐L‐Ara*f*) by mutases before being moved back into the Golgi by UDP‐Ara*f* transporters (UAfTs) where Ara*f* serves as the predominant building block for synthesis of RG‐I α‐1, 5‐arabinan, putatively via Arabinan deficient 1 (ARAD1) (Harholt et al., 2006; Rautengarten et al., 2011; Rautengarten et al., 2017). After assembly in the Golgi, cell wall polymers like pectins are secreted into the apoplast and integrated into the cell wall (Hoffmann et al., 2021). Nucleotide sugars can also be formed from recycled cell wall sugars that are released from cell wall polysaccharides and transported into the cytosol by sugar transport proteins (Barnes & Anderson, 2018). Metabolic salvage of these sugars is achieved via the activities of sugar‐1‐kinases and UDP‐sugar‐pyrophosphorylase (USP) (Geserick & Tenhaken, 2013). Galactose salvage is initiated by phosphorylation through galactokinase (GALK) (Egert et al., 2012). Subsequently, galactose‐1‐phosphate is converted to UDP‐D‐galactose by USP, while conversion to UDP‐D‐glucose seems to be mainly achieved by UGE1 and UGE3 (Barber et al., 2006; Geserick & Tenhaken, 2013).

As the outermost layer of plant cells, cell walls represent an important barrier against phytopathogen infection, and changes in cell wall composition can affect pathogen resistance (Molina et al., 2021). Cell wall‐penetrating fungal pathogens are adapted to the cell wall composition of their hosts by expressing cell wall‐degrading enzymes that allow local cell wall decomposition and fungal entry (for a review see Munzert & Engelsdorf, 2025). The hemibiotrophic ascomycete *Colletotrichum higginsianum* infects Arabidopsis rosettes by penetrating the leaf surface with a specialized infection structure termed appressorium. Appressoria use a combination of turgor pressure and cell wall‐degrading enzyme activity to drive a penetration peg through the epidermal cuticle and cell wall (Ryder et al., 2022). After successful entry around 36 h post‐infection, *C. higginsianum* develops biotrophic hyphae in the leaves, but remains separated from the host cytoplasm by the host plasma membrane (O'Connell et al., 2004). Around 3 days post‐infection, the fungus switches to a necrotrophic lifestyle, leading to lysis of infected cells and colonization of adjacent leaf tissue (Engelsdorf et al., 2013). The entry rate of *C. higginsianum* strongly depends on pectin content and/or composition of the host cell walls, likely explaining enhanced fungal entry into *pgm* leaves (Engelsdorf et al., 2017).

Here, we investigated how carbon starvation leads to dynamic changes of the cell wall during extended nights in wild type and *pgm* rosettes and assessed the importance of cell wall remodeling for resistance to fungal infection. We found that galactose is reversibly released from cell wall galactan upon carbon starvation and that the abundance of cell wall galactan determines the entry rate of *C. higginsianum* under starvation conditions.

## RESULTS

### Starch deficiency and dark‐induced starvation cause similar rosette cell wall phenotypes

**Figure 1:**
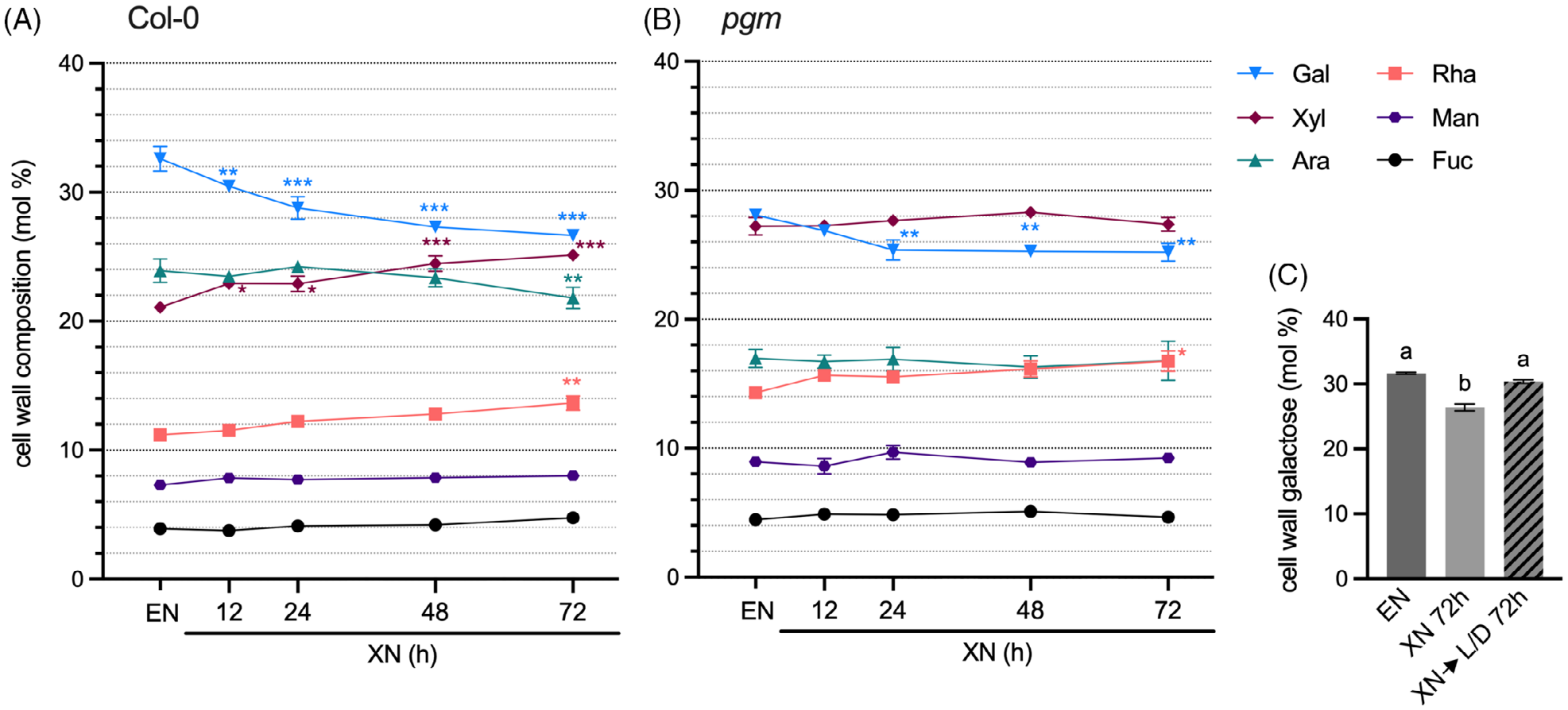
Galactose amount in leaf rosette cell walls is reversibly decreased upon dark‐induced starvation. (A) Col‐0 and (B) *pgm* plants were subjected to an extended night (XN) after a diurnal 12 h light/12 h dark cycle. Whole rosettes were sampled at the end of the regular night (EN) and after 12, 24, 48, and 72 h XN. The relative composition of the neutral cell wall monosaccharides galactose (Gal), xylose (Xyl), arabinose (Ara), rhamnose (Rha), mannose (Man) and fucose (Fuc) is depicted as the molar percentage of total cell wall neutral monosaccharide content (mol %). Values are means (*n* = 4), and error bars represent the SEM. Asterisks indicate statistically significant differences to EN samples for each monosaccharide according to two‐way ANOVA and Dunnett's multiple comparisons test (*P < 0.05, **P < 0.01, ***P < 0.001). (C) Molar percentage of galactose in Col‐0 cell wall neutral monosaccharides at EN, after 72 h XN, and after 72 h XN followed by 72 h in a 12 h/12 h light/dark (L/D) cycle. Values are means (*n* = 4), and error bars represent the SEM. Different letters indicate statistically significant differences according to one‐way ANOVA and Tukey's multiple comparisons test (α = 0.05).

Our previous results suggested that the altered cell wall composition and the reduced abundance of RG‐I side chain epitopes in *pgm* rosettes are caused by periodic nocturnal carbohydrate starvation in diurnal light/dark cycles (Engelsdorf et al., 2017). To test whether dark‐induced starvation in the wild type can phenocopy the cell wall composition of *pgm*, we subjected wild type (Col‐0) and *pgm* plants to an extended night (XN) and sampled rosettes for analyses of the cell wall monosaccharide composition. Starch reserves in the wild type were depleted after 4 h of XN, and soluble sugar content of both genotypes reached a minimum after 12–24 h of XN (Figure S1). In Col‐0, we observed a significantly reduced cell wall galactose content after 12 h XN (Figure 1A). Galactose content was further reduced with prolonged darkness, and at 24–48 h XN, it resembled the galactose content in *pgm* rosette cell walls at the end of the regular night (EN) (Figure 1A,B). After 72 h XN, galactose levels in wild type cell walls were reduced by 18% compared with those before onset of XN (32.6 mol% at EN versus 26.6 mol% at XN 72 h; Figure 1A). While arabinose was also identified as being less abundant in *pgm* cell walls (Engelsdorf et al., 2017), in Col‐0 it was only reduced after 72 h XN and remained more abundant than in *pgm* cell walls (Figure 1A,B). Cell walls of *pgm* rosettes contained significantly less galactose after 24 h of XN compared with EN but exhibited no further decline until 72 h of XN (Figure 1B). Analysis of *gals1/2/3* mutant cell walls showed 23% less galactose compared with Col‐0 and no further reduction upon XN treatment, indicating that only the GALS‐dependent galactose pool is released under dark‐induced starvation (Figure S2). Reduced amounts of galactose were accompanied by a relative increase of xylose (Col‐0) and rhamnose (Col‐0 and *pgm*) during XN progression (Figure 1A,B). Overall, the cell wall monosaccharide composition in Col‐0 showed the highest degree of similarity to *pgm* EN cell walls after 72 h XN (Figure 1A,B). To test whether the starvation‐dependent reduction in cell wall galactose content can be reverted after XN, we subjected Col‐0 plants to 72 h XN before returning them to a regular light/dark cycle. While galactose content after XN was significantly reduced compared with EN, it recovered 72 h after return to a regular light/dark cycle (Figure 1C).

Taken together, dark‐induced starvation in Col‐0 wild type leads to a similar cell wall composition as periodic starvation in rosettes of starch‐deficient *pgm* mutants. Galactose content is significantly but reversibly reduced upon XN treatment, indicating dynamic remodeling of cell wall composition.

### Starvation‐induced β‐galactosidases release galactose from the cell wall

**Figure 2:**
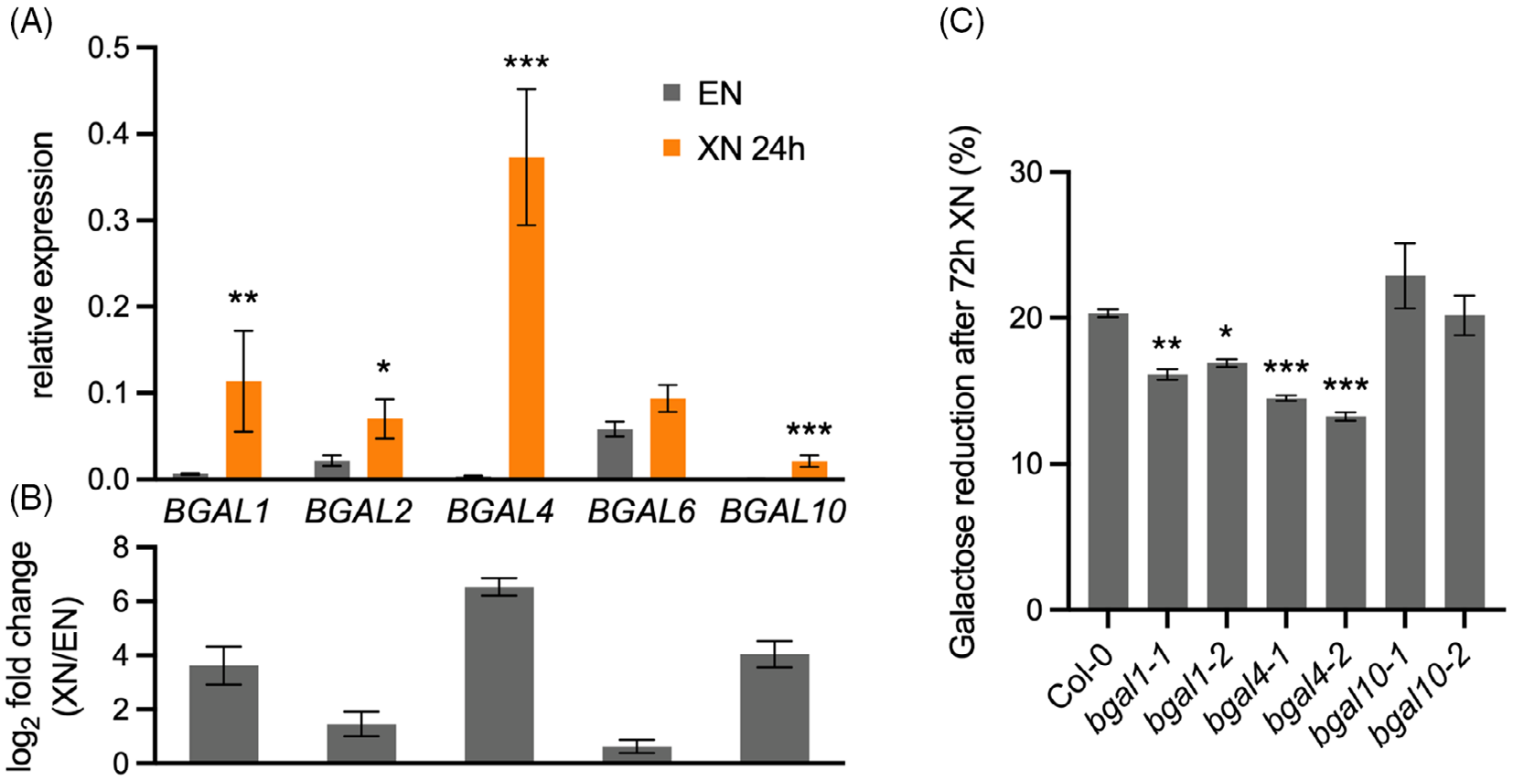
*BGAL1* and *BGAL4* contribute to starvation‐induced release of cell wall galactose. (A) The relative expression of *BGAL1*, *BGAL2*, *BGAL4*, *BGAL6*, and *BGAL10* was determined by quantitative reverse transcriptase polymerase chain reaction (qRT‐PCR) in Col‐0 rosettes at the end of the night (EN) and after 24 h of extended night (XN). Values are means (*n* = 4), and error bars represent the SEM. Asterisks indicate statistically significant differences between XN 24 h and EN according to a Student's *t*‐test (*P < 0.05, **P < 0.01, ***P < 0.001). (B) Bars represent the log_2_ fold change of values depicted in (A) ±SEM. (C) The reduction of galactose in cell walls of Col‐0, *bgal1‐1*, *bgal1‐2*, *bgal4‐1*, *bgal4‐2*, *bgal10‐1*, and *bgal10‐2* rosettes was quantified after 72 h XN relative to EN. Values are means (*n* = 4–5), and error bars represent the SEM. Asterisks indicate statistically significant differences to Col‐0 according to one‐way ANOVA and Dunnett's multiple comparisons test (*P < 0.05, **P < 0.01, ***P < 0.001).

In the light of the known alterations of RG‐I side chains in *pgm* (Engelsdorf et al., 2017), the starvation‐dependent reduction in cell wall galactose suggested that β‐1, 4‐galactan might be remobilized upon carbohydrate shortage. Transcriptomics data by Usadel et al. (2008) indicated that the β‐galactosidases *BGAL1, 2* and *4* are transcriptionally induced in *pgm* during the dark phase, while *BGAL1, 2, 4, 6 and 10* are induced after XN (Table S1). Using quantitative reverse transcriptase polymerase chain reaction (qRT‐PCR), we investigated the induction of these five *BGAL* genes after 24 h of XN, when galactose release was around 50% (cf. Figure 1A). The results confirmed a significant upregulation of *BGAL1, 2, 4* and *10* after XN, while *BGAL6* transcript was not significantly different between XN and control conditions (Figure 2A). *BGAL1, BGAL4* and *BGAL10* exhibited a more than 10‐fold induction after XN compared with EN (Figure 2B). To elucidate the contributions of these three *BGAL* genes to the XN‐induced reduction in cell wall galactose, we examined two independent T‐DNA insertion lines per gene. We identified *bgal1‐1, bgal1‐2, bgal4‐1* and *bgal4‐2* as loss‐of‐function mutants (Figure S3), and obtained *bgal10‐1* and *bgal10‐2*, which have been previously described as xyloglucan β‐galactosidase mutants (Sampedro et al., 2012). An analysis of the galactose content in the rosette cell walls of these mutants after 72 h XN indicated that the decrease in galactose was less pronounced in *bgal1‐1* (16.1% reduction of cell wall galactose content at 72 h XN compared with EN), *bgal1‐2* (16.9%), *bgal4‐1* (14.5%), and *bgal4‐2* (13.2%) compared with Col‐0 (20.3%), while in *bgal10‐1* (22.9%) and *bgal10‐2* (20.2%) XN‐induced galactose release from the cell wall was not impaired (Figure 2C). Mutants for *BGAL1* and *BGAL4* exhibited different degrees of increased galactose contents at EN and XN, indicating additional functions of these enzymes under control conditions (Figure S4).

These results indicate that BGAL1 and BGAL4 contribute to starvation‐dependent galactan degradation, while BGAL10 is not required for the release of galactose from leaf cell walls during XN.

### Galactose salvage is induced upon starvation and cannot be prevented in *uge1 uge3* mutants

**Figure 3:**
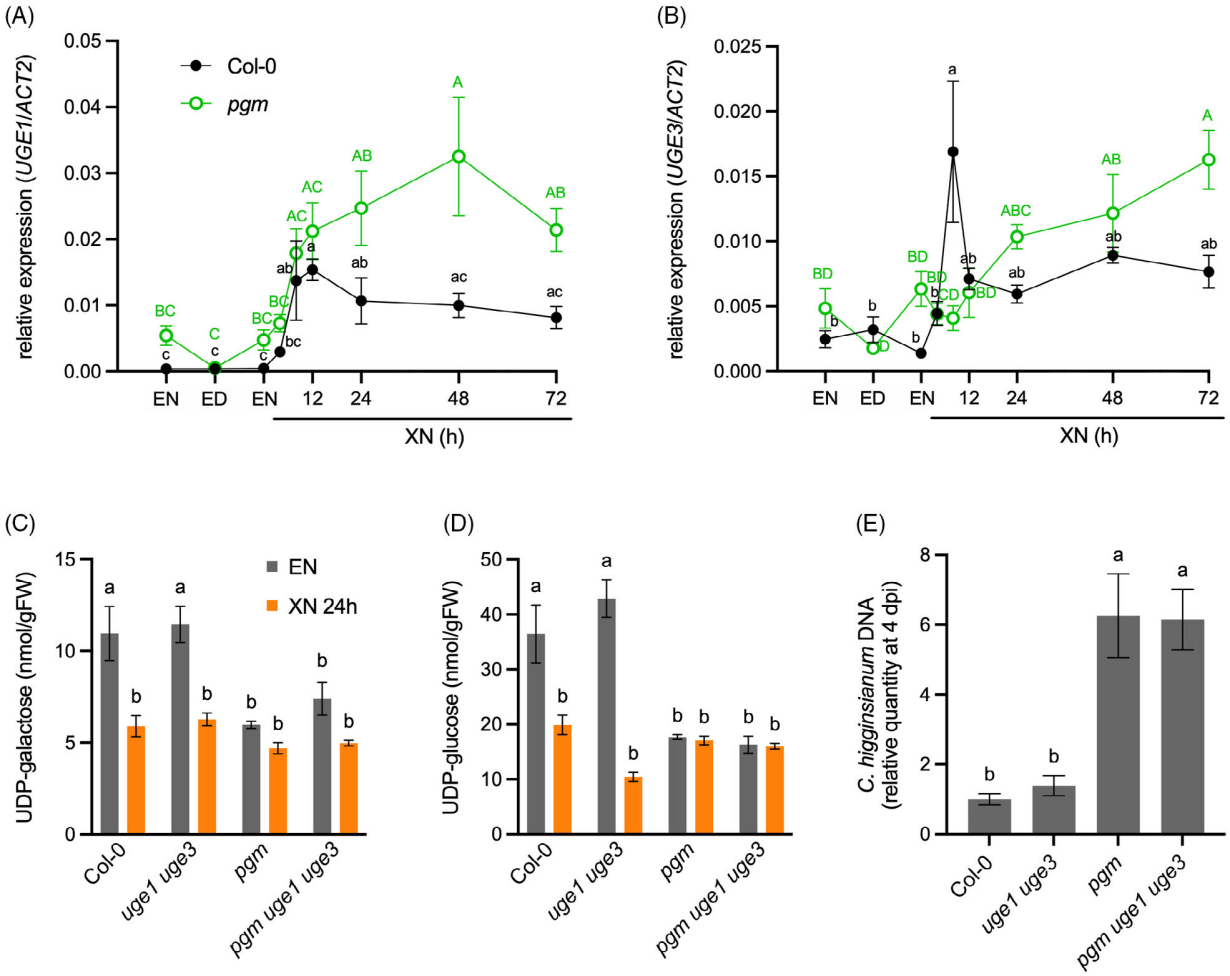
Starvation‐induced *UGE1* and *UGE3* expression is dispensable for galactose salvage. The relative expression of (A) *UGE1* and (B) *UGE3* was determined in rosette leaves of Col‐0 (black) and *pgm* (green) via quantitative reverse transcriptase polymerase chain reaction (qRT‐PCR) analysis at the end of the night (EN) and the end of the day (ED) in a 12 h light/12 h dark cycle, as well as after an extended night (XN) of 4, 8, 12, 24, 48, and 72 h. Values are means (*n* = 3–4), and error bars represent the SEM. Different letters indicate statistically significant differences according to one‐way ANOVA and Tukey's multiple comparisons test for Col‐0 (black) and *pgm* (green, capital letters) samples, respectively (α = 0.05). (C) UDP‐galactose and (D) UDP‐glucose were quantified in Col‐0, *uge1 uge3*, *pgm*, and *pgm uge1 uge3* rosettes at EN and 24 h XN. Values are means (*n* = 5), and error bars represent the SEM. Different letters indicate statistically significant differences according to two‐way ANOVA and Tukey's multiple comparisons test (*α* = 0.05). (E) Rosettes of the indicated genotypes were spray‐infected with *Colletotrichum higginsianum*, and the fungal proliferation was assessed via quantification of fungal DNA via qPCR at 4 days post‐infection (dpi). Values are means (*n* = 6), and error bars represent the SEM. Different letters indicate statistically significant differences according to one‐way ANOVA and Tukey's multiple comparisons test (*α* = 0.05).

Published transcriptomics data indicated an induction of the galactan salvage pathway after XN in the wild type and in *pgm* at EN (Engelsdorf et al., 2017; Usadel et al., 2008). We hypothesized that galactose salvage might be important to provide sugars to rosette leaves under starvation, while at the same time galactose release from the cell wall might impair the cell wall structure and penetration resistance to fungal infection. To further characterize the transcriptional activation of the salvage pathway, we investigated the expression of *UGE1* and *UGE3*, which have been proposed to act in carbohydrate catabolism (Barber et al., 2006), via qRT‐PCR. In both Col‐0 and *pgm*, XN led to a significant increase in *UGE1* expression (Figure 3A). *UGE3* transcripts showed a similar pattern but had a lower expression level than *UGE1* (Figure 3B). Since galactose released from the cell wall via BGAL activity has to pass through the salvage pathway for re‐utilization by the plant, these data suggested that the metabolic flux through the salvage pathway might be increased compared with carbon‐replete wild type plants under control conditions. To investigate whether loss of UGE1 and UGE3 causes a backlog of UDP‐galactose upon starvation, we crossed *pgm* with *uge1 uge3* double mutants and investigated nucleotide sugar levels at EN and after XN. Surprisingly, UDP‐galactose amount was neither increased in *uge1 uge3* rosettes compared with the wild type upon starvation, nor in *pgm uge1 uge3* rosettes compared with *pgm*, indicating that UGE1 and UGE3 are not the only UGE isoforms responsible for galactose salvage (Figure 3C). Instead, the amounts of UDP‐galactose and UDP‐glucose were strongly reduced after XN and in *pgm*, probably reflecting reduced overall carbohydrate availability (Figure 3C,D). The cell wall composition of *uge1 uge3* after 72 h of XN was not altered compared with Col‐0, which excludes the possibility that recycled UDP‐galactose is immediately reused for cell wall synthesis (Figure S5). To investigate whether impaired *UGE1* and *UGE3* expression influences susceptibility of *pgm* to *C. higginsianum*, we infected Col‐0, *uge1 uge3*, *pgm*, and *pgm uge1 uge3* rosettes with the fungus and assessed fungal genomic DNA as a proxy for fungal biomass at 4 days post‐infection (dpi) via qPCR. Susceptibility to *C. higginsianum* was not affected by the loss of *UGE1* and *UGE3* (Figure 3E).

To summarize, transcriptional activation of *UGE1* and *UGE3* indicated increased galactose salvage upon XN and in *pgm*. However, loss of *UGE1* and *UGE3* is neither sufficient to cause UDP‐galactose backlog nor to alter starvation‐dependent pathogen susceptibility, indicating that they do not represent the main catabolic UGE isoforms.

### Increased cell wall galactan limits starvation‐dependent pathogen entry

**Figure 4:**
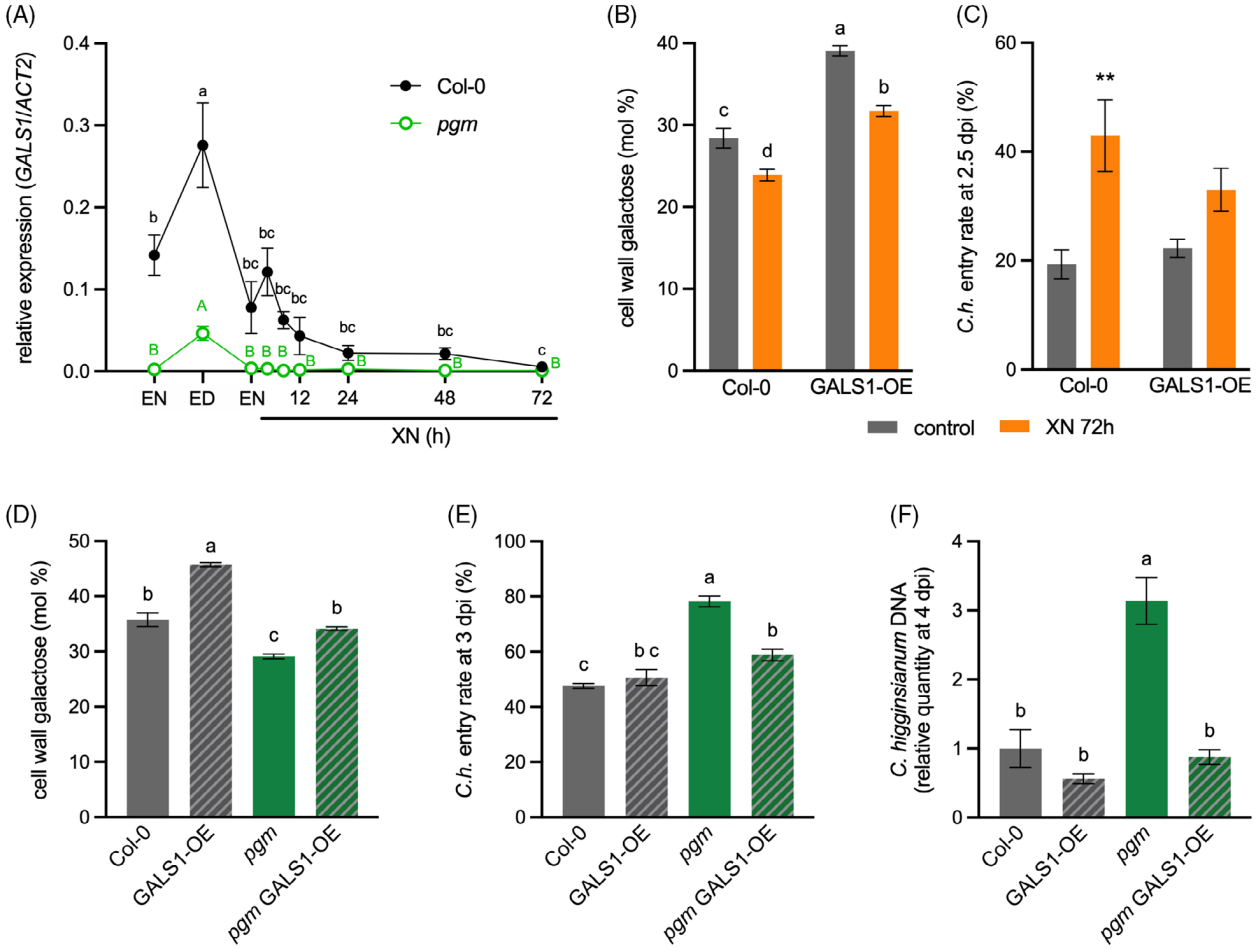
Galactan content in leaf cell walls contributes to resistance to *Colletotrichum higginsianum* infection. (A) The relative expression of *GALS1* was determined in rosette leaves of Col‐0 (black) and *pgm* (green) via quantitative reverse transcriptase polymerase chain reaction (qRT‐PCR) analysis at the end of the night (EN) and the end of the day (ED) in a 12 h light/12 h dark cycle, as well as after 4, 8, 12, 24, 48, and 72 h extended night (XN). Values are means (*n* = 3–4), and error bars represent the SEM. Different letters indicate statistically significant differences according to one‐way ANOVA and Tukey's multiple comparisons test for Col‐0 (black) and *pgm* (green, capital letters) samples, respectively (α = 0.05). (B) Molar percentage of galactose in Col‐0 and GALS1‐OE cell wall neutral monosaccharides under control conditions (EN) and after 72 h XN. Values are means (*n* = 4), and error bars represent the SEM. Different letters indicate statistically significant differences according to two‐way ANOVA and Tukey's multiple comparisons test (α = 0.05). (C) Entry rate of *Colletotrichum higginsianum* at 2.5 days post‐infection (dpi) in Col‐0 and GALS1‐OE under control conditions (EN) and after 72 h XN. Values are means (*n* = 5–7), and error bars represent the SEM. Asterisks indicate statistically significant differences to control conditions according to Šidák's multiple comparisons test (*P < 0.05, **P < 0.01). (D) Molar percentage of galactose in cell wall neutral monosaccharides, (E) *C. higginsianum* entry rate at 3 dpi, and (F) *C. higginsianum* DNA at 4 dpi were quantified in Col‐0, GALS1‐OE, *pgm*, and *pgm* GALS1‐OE. Values are means (D, *n* = 4; E, F, *n* = 5), and error bars represent the SEM. Different letters indicate statistically significant differences according to one‐way ANOVA and Tukey's multiple comparisons test (α = 0.05).

The reduction in cell wall galactose within hours after onset of starvation, together with published transcriptomics data indicating attenuated *GALS1* expression, suggested that galactan synthesis might be reduced in addition to the mobilization of galactose from existing galactan chains (Engelsdorf et al., 2017; Usadel et al., 2008). A qRT‐PCR time‐course analysis of *GALS1* confirmed increased expression at the end of the day (ED) and strongly reduced *GALS1* transcript amounts at EN and after XN (Figure 4A). *GALS1* transcripts were depleted in *pgm* at EN and in Col‐0 after 72 h XN (Figure 4A).

*GALS1* overexpressing lines (GALS1‐OE) exhibit about 200‐fold increased *GALS1* expression and high β‐1, 4‐galactan levels (Figure S6; Liwanag et al., 2012). We employed these GALS1‐OE lines to test the hypothesis that an increased galactan content in rosette cell walls might protect against starvation‐dependent cell wall defects and associated susceptibility toward *C. higginsianum*. We confirmed that *GALS1* overexpression was not affected by 72 h of XN (Figure S6). GALS1‐OE cell walls showed intact galactose release after XN, but galactose amounts remained significantly higher than in non‐starved Col‐0 rosettes (Figure 4B). To test whether the higher galactose content influences penetration resistance to *C. higginsianum*, we determined the fungal entry rate. After 72 h XN compared with control conditions, fungal entry was significantly increased in Col‐0, but not in GALS1‐OE, suggesting that an increased cell wall galactan content mitigates the penetration success of the fungus (Figure 4C). Comparison of fungal entry rates in Col‐0 after XN and *pgm* under control conditions indicated that dark‐induced and periodic starvation led to a similar impairment of penetration resistance (Figure S7). As the cell wall composition of *pgm* rosettes resembles that of Col‐0 after XN, we hypothesized that *GALS1* overexpression would also reduce penetration success of *C. higginsianum* in the *pgm* background. We crossed *pgm* with GALS1‐OE plants and confirmed that *GALS1* expression and galactose content were increased in the resulting line (Figure 4D, Figure S6b). In both Col‐0 and *pgm* rosettes, *GALS1* overexpression led to an altered composition of neutral cell wall monosaccharides, with an increased molar ratio of galactose, whereas the major pectic sugar galacturonic acid was not affected (Figure S8). The fungal entry rate in *pgm* GALS1‐OE was significantly reduced compared with *pgm*, indicating increased penetration resistance (Figure 4E). Galactan‐dependent resistance of *pgm* GALS1‐OE to *C. higginsianum* was also detected via qPCR of fungal DNA at 4 dpi, demonstrating that the reduced entry rate leads to reduced fungal proliferation and a suppression of *pgm* hypersusceptibility (Figure 4F).

**Figure 5:**
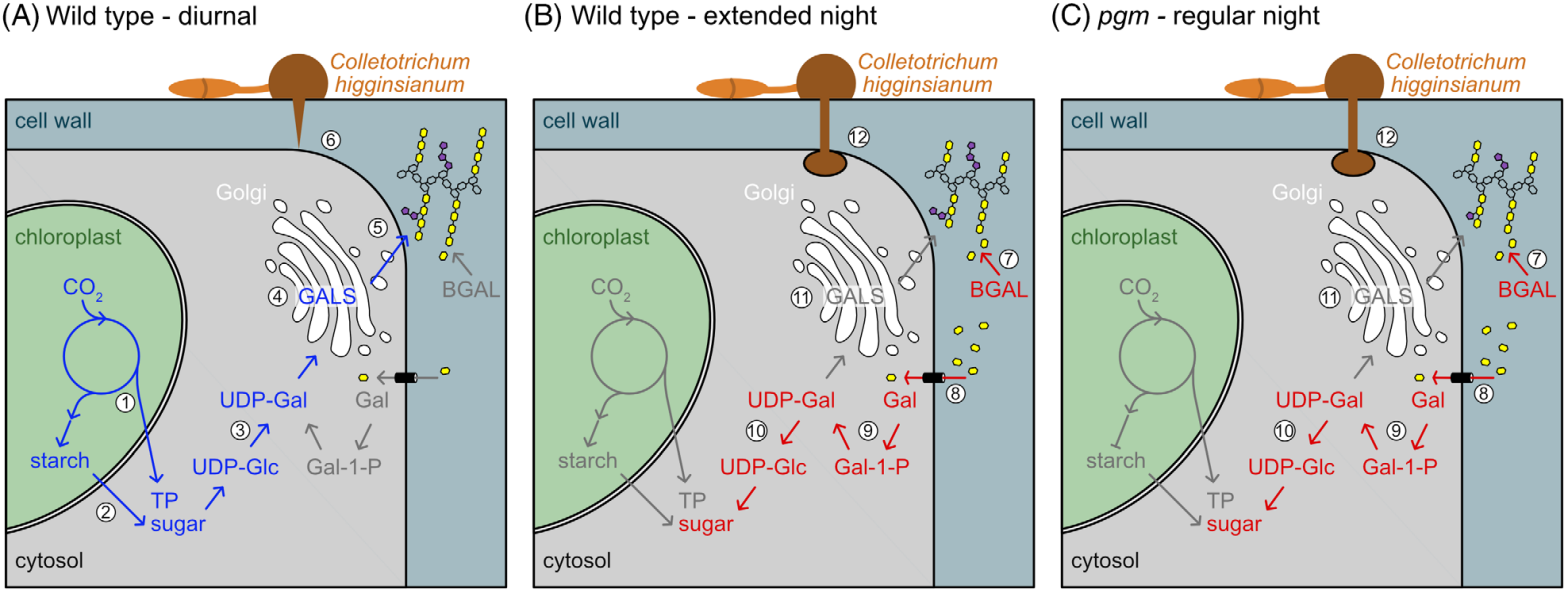
Model illustrating cell wall galactan remodeling upon starvation and its impact on fungal penetration resistance. (A) Carbon usage for galactan synthesis and penetration resistance in Arabidopsis wild type rosettes during a regular diurnal cycle: (1) Carbon fixed in the Calvin cycle during the day is exported to the cytosol as triose phosphate (TP) and stored in the form of starch as a reservoir for the night. (2) Starch is degraded to maltose and glucose during the night, and sugars are utilized for UDP‐glucose (UDP‐Glc) synthesis in the cytosol. (3) UDP‐galactose (UDP‐Gal) is formed from UDP‐Glc via the action of UDP‐Glc 4‐epimerases (UGEs). (4) After import into the Golgi, UDP‐Gal serves as substrate for β‐1,4‐galactan synthesis by Galactan synthase (GALS). (5) Galactan is secreted into the apoplast and linked to pectic rhamnogalacturonan‐I (RG‐I). (6) An intact cell wall limits penetration by the hemibiotrophic fungus *Colletotrichum higginsianum*. Cell wall galactan recycling (B) during an extended night or (C) during a regular night in starch‐deficient *phosphoglucomutase* (*pgm*) mutants: (7) β‐galactosidase (BGAL) expression is induced upon starvation and leads to the release of Gal from RG‐I galactan chains (yellow sugar symbols). *pgm* mutants exhibit a reduced arabinose amount (purple sugar symbols). (8) Gal is imported to the cytosol via sugar transporters. (9) The Gal salvage pathway converts Gal to Gal‐1‐P and UDP‐Gal. (10) UGEs epimerize UDP‐Gal to UDP‐Glc, which can be utilized for cellular metabolism. (11) GALS1 expression is strongly downregulated, contributing to a decreasing galactan amount in the cell wall and (12) impaired penetration resistance against *C. higginsianum*.

Collectively, our data show that carbohydrate starvation causes the release of galactose from pectic β‐1, 4‐galactan via BGAL1 and BGAL4 activities, while reduced *GALS1* expression prevents the flux of carbon into galactan biosynthesis (Figure 5). The resulting alteration in cell wall composition critically affects penetration resistance against *C. higginsianum*, which can be rescued by GALS1 overexpression.

## DISCUSSION

Structural carbohydrates of the cell wall represent the largest carbon sink in plants (Barnes & Anderson, 2018). Previous reports indicated that dark‐induced starvation leads to an induction of glycoside hydrolases and an altered cell wall composition (Lee et al., 2007; Poschet et al., 2010). In Arabidopsis mutants with impaired starch synthesis or mobilization, cell wall arabinose and galactose levels were reduced compared with the wild type, indicating that periodic carbohydrate shortage in these lines influences polysaccharide biosynthesis and/or degradation (Engelsdorf et al., 2017). However, to date it was not known to what degree carbon can be remobilized from cell walls, which cell wall polymers would be prioritized for recycling, and how this remodeling influences the cell wall's function in pathogen resistance.

Here, we undertook a detailed comparison of the cell wall monosaccharide composition in rosettes of wild type and the starch‐deficient *pgm* mutant upon dark‐induced starvation. Our data reveal that galactose is the major sugar released from cell walls under these conditions. Of note, the galactose levels in *pgm* cell walls under control conditions resembled those of the wild type after 2 days of starvation. This is in line with previously published transcript and metabolite datasets showing similarities between carbon‐limited *pgm* and XN‐starved wild type leaves (Gibon et al., 2006; Usadel et al., 2008). In *pgm*, galactose content was not further reduced after 2 days of XN, indicating that it cannot be reduced below approximately 25% of the total neutral cell wall monosaccharides. Indeed, the *gals1/2/3* triple mutant lacking all three GALS isoforms has a similar galactose content as the starved *pgm* mutant. As no additional galactose is released from the cell walls of *gals1/2/3* mutants upon XN, we speculate that carbon depletion induces degradation of galactan side chains in RG‐I. Arabinan side chains in RG‐I seem to be less affected by dark‐induced starvation. While arabinose is also reduced in cell walls of starch‐deficient mutants (Engelsdorf et al., 2017), it was only mildly altered up to 48 h XN in wild type rosettes. These data indicate that the low arabinose content in the *pgm* mutant is less connected to starvation. Altered galactan content in *gals1/2/3* and GALS1‐OE lines does not cause obvious visible phenotypes (Ebert et al., 2018), suggesting that RG‐I galactan may represent a suitable and flexible source of carbon to be recycled from the cell wall pool. Consistent with this model, we found that the galactose levels in wild type cell walls can be almost fully recovered after 3 days of returning starved plants to a regular light/dark cycle.

We identified four β‐galactosidase genes that were significantly upregulated after 24 h of XN. Mutant analysis demonstrated that at least two of them, *BGAL1* and *BGAL4*, contribute to the decline of galactose in leaf cell walls during XN. This finding corroborates a previous report that BGAL4 is secreted upon carbohydrate starvation in detached leaves (Lee et al., 2007). Seven additional BGAL isoforms have been reported to be expressed in leaves (Ahn et al., 2007), of which *BGAL2*, *BGAL6*, and *BGAL10* showed upregulation after starvation (Poschet et al., 2010; Usadel et al., 2008). BGAL10 has been shown to represent the main BGAL isoform acting on xyloglucan (Sampedro et al., 2012). As we did not observe a reduced release of galactose in *bgal10* mutants after XN, our data indicate that galactose released upon starvation does not originate from xyloglucan. This is in line with the GALS‐dependency of the released galactose pool and suggests that BGAL1 and BGAL4 degrade RG‐I galactan side chains upon XN treatment. Further studies are required to resolve the contributions of different BGAL isoforms to starvation‐induced galactose release. Beyond their role for carbon metabolism, BGALs may be central to the outcome of pathogen infections by determining the structure of RG‐I side chains and consequently cell wall mechanics. However, the quantitative decrease in galactose release observed with *bgal* single mutants indicates functional redundancy that remains to be investigated.

Released galactose is likely taken up from the cell wall by monosaccharide transporters of the STP family (Barnes & Anderson, 2018; Poschet et al., 2010). Galactose can be recycled for cellular metabolism via a salvage pathway, which involves phosphorylation via GALK and conversion into UDP‐galactose by USP (Egert et al., 2012; Geserick & Tenhaken, 2013; Reiter, 2008). Arabidopsis encodes five UGE isoforms that can interconvert UDP‐galactose and UDP‐glucose. In line with the previously made suggestion that the UGE1 and UGE3 isoforms are mainly acting in carbohydrate catabolism (Barber et al., 2006), we detected a strong upregulation of *UGE1* and *UGE3* upon nocturnal starvation in *pgm* and upon XN, while none of the other *UGE* isoforms was differentially expressed under both conditions (Engelsdorf et al., 2017; Usadel et al., 2008). However, loss of *UGE1* and *UGE3* did not cause differences in UDP‐galactose or UDP‐glucose levels in wild type or *pgm* backgrounds at EN or after XN, which suggests that they can be functionally replaced by the remaining three UGE isoforms. Congruently, *pgm uge1 uge3* mutants were not altered in their susceptibility to *C. higginsianum* infection, leaving open the question of whether remobilized sugars contribute to pathogen defense after uptake from the apoplast. We refrained from further analysis of higher order UGE mutants, as those have been reported to have extreme dwarf phenotypes (Rösti et al., 2007). Nevertheless, it will be exciting to further explore the specific functions of the individual UGE isoforms and their contributions to galactose salvage in the future.

In addition to the induction of BGAL expression and of the galactose salvage pathway, XN treatment led to the downregulation of the galactan synthase genes *GALS1*, *GALS2*, and *GALS3* (Engelsdorf et al., 2017, Usadel et al., 2008). All three GALS isoforms contribute to galactan synthesis *in vivo*, with GALS1 and GALS3 being the main isoforms expressed in leaves, and the loss of GALS1 having the strongest effect on galactose content in leaf cell walls (Ebert et al., 2018; Liwanag et al., 2012). Of note, *GALS1* expression is close to the detection limit in *pgm* leaves already at EN, indicating that galactan synthesis is halted in *pgm* every night when soluble sugar reserves are depleted. The gene expression patterns of *GALS1/2/3*, *UGE1/3*, and *BGAL1/4* together indicate that galactan synthesis and degradation are inversely regulated and balanced depending on the available carbohydrate budget. Our investigation of GALS1‐OE lines showed that the relative amount of galactose present in cell walls of rosette leaves can be increased by more than 30% and remains higher in GALS1‐OE than in Col‐0 (at EN) even after 72 h of XN treatment. Nevertheless, the additional galactose in GALS1‐OE cell walls is amenable to starvation‐induced release. By contrast, reduced galactose levels in *gals1/2/3* triple mutants were not further reduced upon XN, indicating that only RG‐I galactan side chains are recycled upon starvation. Based on our findings, we propose that the galactan side chains in RG‐I represent a dynamic carbohydrate reserve in leaf cell walls that can be utilized upon starvation.

The cell wall represents a major penetration barrier to fungal infection, and infection success often depends on cell wall modifications caused by both the host plant and the pathogen (Munzert & Engelsdorf, 2025). Cell wall polysaccharides are targeted by a wide variety of pathogen‐derived cell wall‐degrading enzymes that are inextricably linked with pathogen lifestyle and host range (Kubicek et al., 2014). To date, few studies have addressed local cell wall remodeling during pathogen infection. Pioneering studies on *Pseudomonas spp*. infections in Arabidopsis and bean revealed local changes in cell wall composition, including the increase of RG‐I galactan and of other pectic polymers (De la Rubia et al., 2023; Kim et al., 2023). We have previously reported that periodic nocturnal starvation in starch‐free mutants leads to reduced arabinose and galactose content and reduced detection of RG‐I/arabinogalactan epitopes in rosette leaf cell walls (Engelsdorf et al., 2017). Comparative infection experiments with other mutants defective in cell wall composition indicated that RG‐I composition (in *mur8*; Mertz et al., 2012) and total pectin abundance (in *pmr5 pmr6*; Vogel et al., 2004) are critical for penetration resistance against *C. higginsianum* (Engelsdorf et al., 2017). In contrast, specific impairment of arabinose (*arad1, mur4*) and galactose (*gals1*) content in cell walls did not influence pathogen susceptibility during a regular diurnal cycle (Engelsdorf et al., 2017). Here, we show that galactose release from rosette cell walls critically affects penetration resistance in plants that suffer from periodic (*pgm*) or dark‐induced (XN) starvation. While sugars derived from photosynthetic carbon assimilation can be utilized for galactan synthesis throughout a regular diurnal cycle (Figure 5A), starvation causes a pause in sugar supply from the chloroplast. Under these conditions, BGALs, STPs, and UGEs are induced to initiate recycling of cell wall galactose via the galactose salvage pathway (Figure 5B,C; Usadel et al., 2008). As a consequence of starvation‐dependent alterations in cell wall composition, penetration resistance against *C. higginsianum* is impaired, leading to an increased fungal entry rate. Our data reveal that overexpression of GALS1, counteracting the starvation‐induced reduction of cell wall galactose, is sufficient to suppress the increased fungal entry rates upon starvation and the hypersusceptibility of *pgm*.

In summary, we present evidence that galactose is recycled from RG‐I side chains in Arabidopsis leaf cell walls during carbohydrate shortage, enabling the plant to tap into cell wall sugar reserves. Due to the highly dynamic organization of pectic polymers (Anderson & Pelloux, 2025), they might be a preferred substrate for sugar recycling. However, the composition of pectin is also of crucial importance for fungal penetration resistance, which is consequently compromised under starvation conditions. This trade‐off between sugar supply for plant metabolism and preformed pathogen defense highlights the importance of cell wall surveillance to sense and maintain functional integrity under stress conditions.

## MATERIALS AND METHODS

### Plant material and growth conditions

*Arabidopsis thaliana* plants were grown in a 12 h light (22°C)/12 h dark (20°C) cycle at a photon flux density of 110 μmol m^−2^ s^−1^. Genotypes used in this study are listed in Table S2. Five days prior to an infection with *Colletotrichum higginsianum* or extended night, plants were fertilized with 40 mL 0.1% Wuxal Super (Aglukon) fertilizer per pot. Five‐week‐old plants were used for starvation and infection experiments.

### Colletotrichum higginsianum infection assays

*Colletotrichum higginsianum* isolate MAFF 305635 (Ministry of Forestry and Fisheries, Japan) was grown on oatmeal agar plates (5% [w/v] shredded oatmeal, 1.2% [w/v] agar) for 7 days in a 16 h light (22°C)/8 h dark (20°C) cycle at a photon flux density of 110 μmol m^−2^ s^−1^. Conidia were harvested by rinsing plates with deionized water; the conidia titer was adjusted to 2 × 10^6^ conidia mL^−1^ and the suspension was immediately used for infection experiments. Spray infection was performed as described by Voll et al. (2012). Briefly, inoculation was performed at the end of the light phase, and high humidity was maintained until 2.5 days post‐infection to provide consistent conditions for humidity‐dependent fungal penetration. Fungal entry rates were determined under the microscope after staining fungal structures with trypan blue, as described (Engelsdorf et al., 2017). Quantification of the relative fungal DNA content in infected leaves was performed by qPCR analysis of *Ch*TrpC, as previously described (Engelsdorf et al., 2013).

### Gene expression analysis

Total RNA was isolated using a NucleoSpin Plant and Fungi RNA Mini Kit (Macherey‐Nagel). One microgram of total RNA was treated with RNase‐Free DNase (Thermo Scientific) and complementary DNA (cDNA) synthesized with a RevertUP II Reverse Transcriptase Kit (Biotechrabbit). Quantitative reverse transcription polymerase chain reaction was performed using Blue S'Green (Biozym) or PowerUp (Applied Biosystems) SYBR green mix and a Bio‐Rad CFX Connect RT‐PCR system. Primers are listed in Table S3 and were diluted according to the manufacturer's specifications. *ACT2* was used as a reference in all experiments.

### Quantification of soluble sugars and starch

Soluble sugars and starch were analyzed as described by Voll et al. (2003). Briefly, snap‐frozen leaves were extracted twice with 80% ethanol at 80°C. After evaporation of ethanol and resuspending in deionized water, soluble glucose, fructose, and sucrose were quantified in a coupled enzymatic assay using a Tecan infinite 200Pro microtiter plate reader. Extracted leaves were ground in 0.2 M KOH, and starch was solubilized by heating for 45 min at 95°C. After degradation by a mixture of α‐amylase and amyloglucosidase, resulting glucose was quantified as described above.

### UDP‐galactose and UDP‐glucose analysis

The extraction of UDP‐galactose and UDP‐glucose was done as described previously (Rautengarten et al., 2019). Briefly, whole Arabidopsis rosettes were ground in liquid nitrogen and extracted in ice‐cold chloroform/methanol (3:7). After incubation for 2 h at −20°C, ice‐cold water was added, and the upper phase was collected after 5 min centrifugation at 4°C and 30,000*g*. Extraction was repeated two times, and combined extracts were lyophilized. Ten millimoles of ammonium bicarbonate was added to the dried sample, mixed, and briefly spun down. Equilibration of an EnviCarb SPE (Sigma‐Aldrich) was performed using 80% acetonitrile with 0.1% trifluoroacetic acid (TFA) followed by 2 mL of water. The sample in 10 mM ammonium bicarbonate was added to the SPE column, and the column was washed with water, 25% acetonitrile, and finally 50 mM triethylamine/acetic acid (TEAA) pH 7.0. Elution was done with 25% acetonitrile with 50 mM TEAA pH 7.0. Following an overnight lyophilization step, the UDP‐sugars were resuspended in ice‐cold water and analyzed by LC–MS/MS. LC–MS/MS was performed using porous graphitic carbon as the stationary phase on an 1100 series HPLC system (Agilent Technologies) and a 4000 QTRAP LC/MS/MS system (SCIEX) equipped with a TurboIonSpray ion source using methods previously described (Rautengarten et al., 2014).

### Cell wall analysis

Whole Arabidopsis rosettes were sampled at the end of the night (EN) or after 12, 24, 48, and 72 h of extended night (XN) as indicated in the figure legends. Samples were ground in liquid nitrogen and extracted three times with 80% ethanol at 80°C and washed with deionized water. Alcohol‐insoluble residue (AIR) was dried by lyophilization, weighed in 2‐mL tubes with screw caps, and used for cell wall hydrolysis as described by Yeats et al. (2016). Briefly, neutral cell wall sugars and uronic acids from non‐crystalline cell wall matrix polymers were hydrolyzed in 4% (w/v) sulfuric acid by autoclaving at 121°C for 60 min. Monosaccharides were diluted with ultrapure water, and ribose was added as an internal standard. Analysis was performed via high‐performance anion‐exchange chromatography with pulsed amperometric detection (HPAEC‐PAD) using a biocompatible Knauer Azura HPLC system and an Antec Decade Elite SenCell detector heated to 40°C. Monosaccharides were separated on a Thermo Fisher Dionex CarboPac PA20 BioLC analytical column (3 × 150 mm) equipped with a CarboPac PA20 BioLC guard column (3 × 30 mm) using the following solvent gradient of (B) 10 mM NaOH and (C) 700 mM NaOH in (A) water, at a 0.4 mL/min flow rate: 0–25 min: 20% B; 25–28 min: 20% to 0% B, 0%–70% C; 28–33 min: 70% C; 33–35 min: 70% to 100% C; 35–38 min: 100% C; 38–42 min: 0%–20% B, 100% to 0% C; 42–60 min: 20% B.

The cell wall neutral monosaccharide composition represents the molar percentage of fucose, rhamnose, arabinose, galactose, xylose and mannose in relation to the sum of all neutral monosaccharides. The relative reduction in cell wall galactose amount under XN conditions was calculated for individual replicates of XN treated samples using the following formula:

$$100-\left(\frac{\text{galactose}\;\mathrm{XN}}{\text{mean}\;\text{galactose}\;\mathrm{EN}}\times 100\right).$$

### Statistical analysis

Statistical analysis was performed with GraphPad Prism (Version 10.4.0). Specific statistical methods, sample size, significance level, and *P*‐values are indicated in the respective figure legends.

## Author Contributions

Conceptualization and writing—original draft: TE. Investigation: LR, RNM, KSM‐E, ST, CR, VT, KLH, LZ, and TE. Funding acquisition: BE and TE. Supervision: RNM, KSM‐E, BE, and TE. Writing—review and editing: LR, RNM, KSM‐E, ST, CR, VT, KLH, LZ, BE, TE.

## Conflict of Interest

The authors declare no conflicts of interest.

## Supporting information


**Figure S1.** Carbohydrate depletion during dark‐induced starvation in leaves.
**Figure S2.** Galactose content in *gals1/2/3* mutants.
**Figure S3.** Characterization of *bgal1* and *bgal4* mutants.
**Figure S4.** Cell wall galactose amount in *BGAL1, BGAL4*, and *BGAL10* mutants.
**Figure S5.** Cell wall monosaccharide composition in *uge1 uge3* rosettes upon starvation.
**Figure S6.** *GALS1* expression in GALS1‐OE lines.
**Figure S7.** Dark‐induced and periodic starvation lead to increased entry of *Colletotrichum higginsianum*.
**Figure S8.** Cell wall monosaccharide composition in rosettes of GALS1‐OE lines.
**Table S1.** Expression of *BETA‐GALACTOSIDASE* (*BGAL*) genes upon periodic and dark‐induced starvation.
**Table S2.** Arabidopsis genotypes used in this study.
**Table S3.** Primers used in this study.

## Data Availability

The data that supports the findings of this study are available in the supplementary material of this article.
