# Supplementary material for: Galactan mobilization during carbon starvation compromises plant cell wall‐mediated resistance to fungal infection
Source: Plant J. 2025 Aug 21;123(4):e70438. doi: 10.1111/tpj.70438 (PMC12370242; doi:10.1111/tpj.70438)
Supplement: Supplementary file 1 — Figure S1. Carbohydrate depletion during dark‐induced starvation in leaves. Figure S2. Galactose content in gals1/2/3 mutants. Figure S3. Characterization of bgal1 and bgal4 mutants. Figure S4. Cell wall galactose amount in BGAL1, BGAL4, and BGAL10 mutants. Figure S5. Cell wall monosaccharide composition in uge1 uge3 rosettes upon starvation. Figure S6. GALS1 expression in GALS1‐OE lines. Figure S7. Dark‐induced and periodic starvation lead to increased entry of Colletotrichum higginsianum. Figure S8. Cell wall monosaccharide composition in rosettes of GALS1‐OE lines. Table S1. Expression of BETA‐GALACTOSIDASE (BGAL) genes upon periodic and dark‐induced starvation. Table S2. Arabidopsis genotypes used in this study. Table S3. Primers used in this study. [file TPJ-123-0-s001.docx]

**Supporting Information**


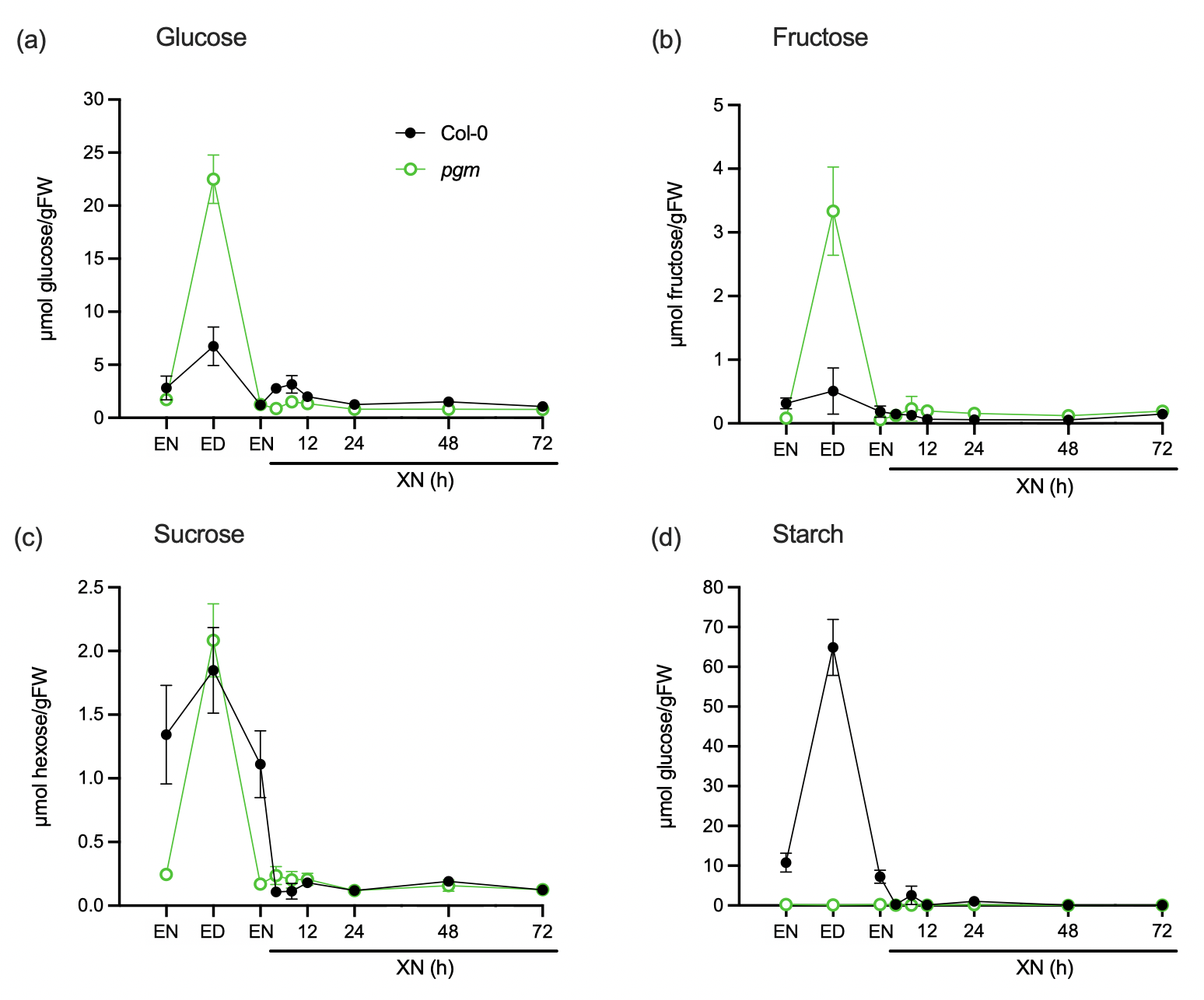


**Figure S1: Carbohydrate depletion during dark-induced starvation in leaves.**

(a) Glucose, (b) fructose, (c) sucrose and (d) starch were quantified in rosette leaves of Col-0 (black) and *pgm* (green) at the end of the night (EN) and the end of the day (ED) in a 12h light / 12h dark cycle, as well as after 4, 8, 12, 24, 48 and 72h extended night (XN). Values are means (n=4) and error bars represent the SEM.

**
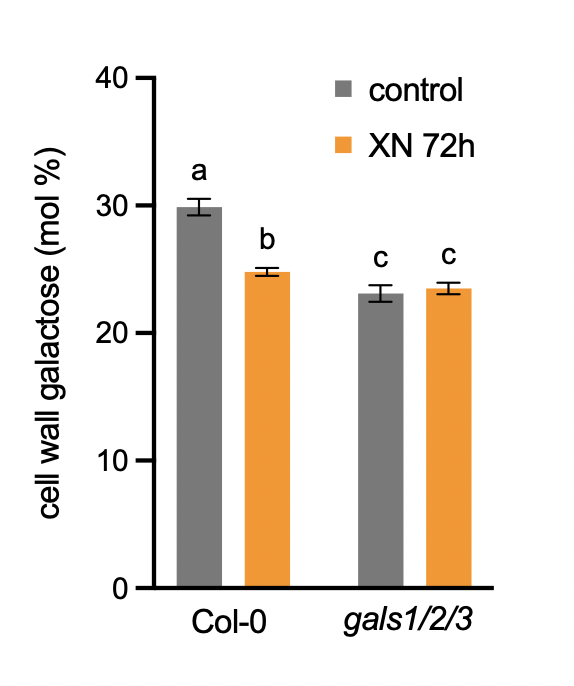
**

**Figure S2: Galactose content in *gals1/2/3* mutants.**

Molar percentage of galactose in cell wall neutral monosaccharides in Col-0 and *gals1/2/3* under control conditions (EN) and after 72h XN. Values are means (n=3-4) and error bars represent the SEM. Different letters indicate statistically significant differences according to two-way ANOVA and Tukey's multiple comparisons test (α = 0.05).

**
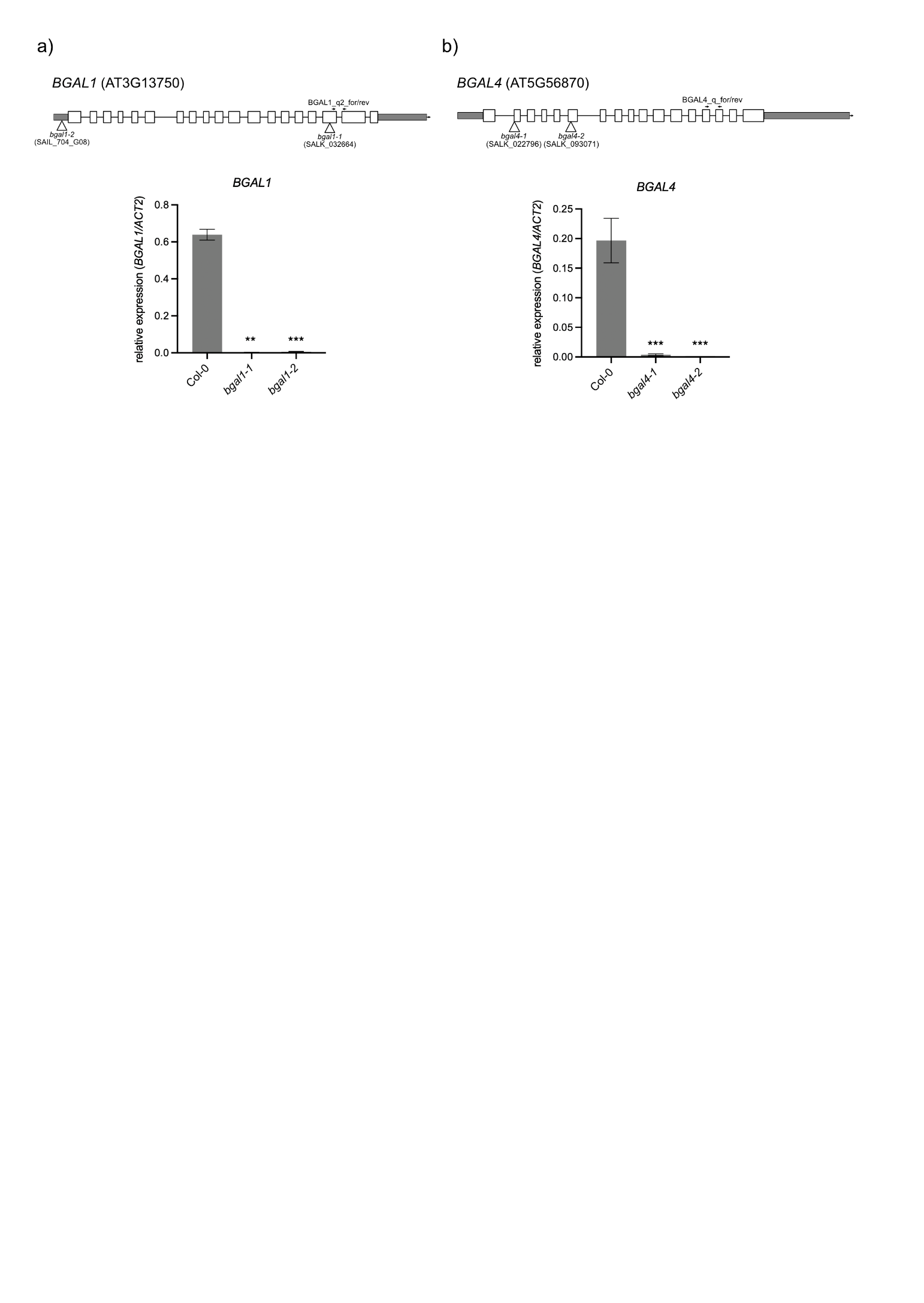
**

**Figure S3: Characterization of *bgal1* and *bgal4* mutants.**

The relative expression of (a) *BGAL1* in Col-0, *bgal1-1* and *bgal1-2* rosettes and (b) *BGAL4* in Col-0, *bgal4-1* and *bgal4-2* rosettes were determined by qRT-PCR after 24h of extended night. Sketches of the genes indicate exons (white boxes), introns (lines), UTR regions (grey boxes), T-DNA insertion sites (triangles), and primer binding sites (arrows). Values are means (n=4) and error bars represent the SEM. Asterisks indicate statistically significant differences to Col-0 according to a Student’s t-Test (**p<0.01, ***p<0.001).


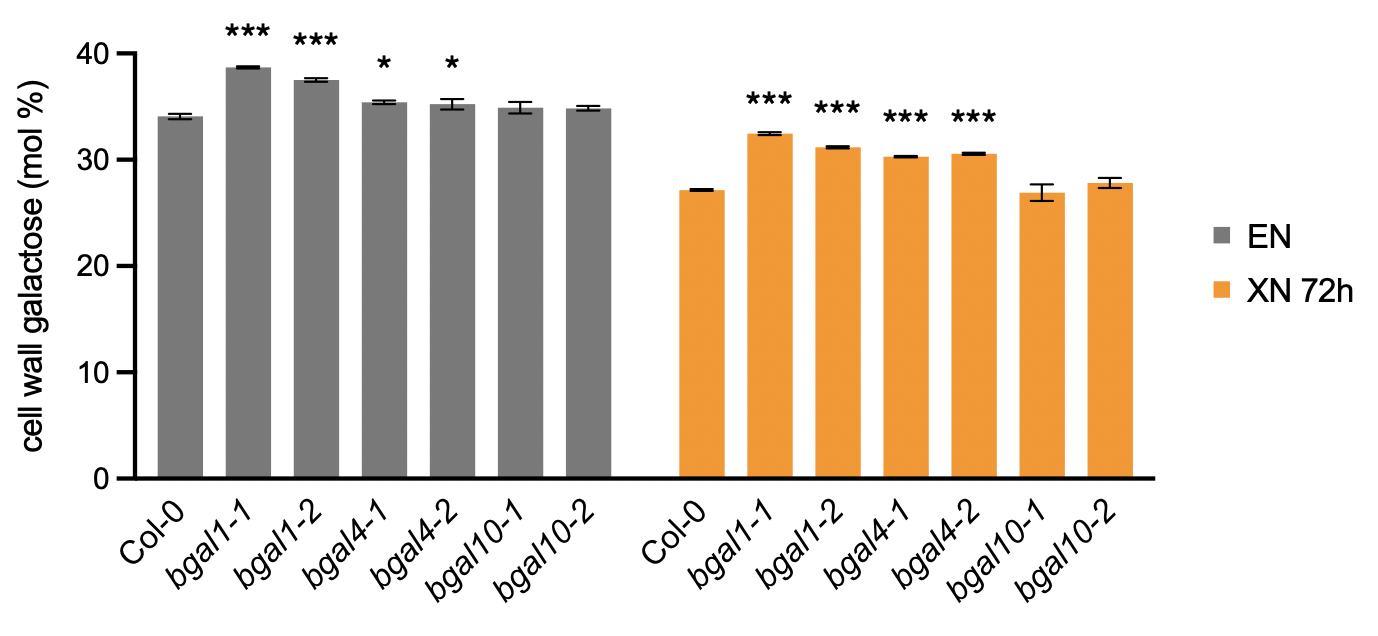


**Figure S4: Cell wall galactose amount in *BGAL1, BGAL4* and *BGAL10* mutants.**

The molar percentage of galactose in cell wall neutral monosaccharides was determined in Col-0, *bgal1-1*, *bgal1-2*, *bgal4-1*, *bgal4-2*, *bgal10-1* and *bgal10-2* rosettes at the end of the night (EN) and after 72h extended night (XN). Values are means (n=4-5) and error bars represent the SEM. Asterisks indicate statistically significant differences to Col-0 according to two-way ANOVA and Dunnett's multiple comparisons test (*p<0.05, **p<0.01, ***p<0.001).


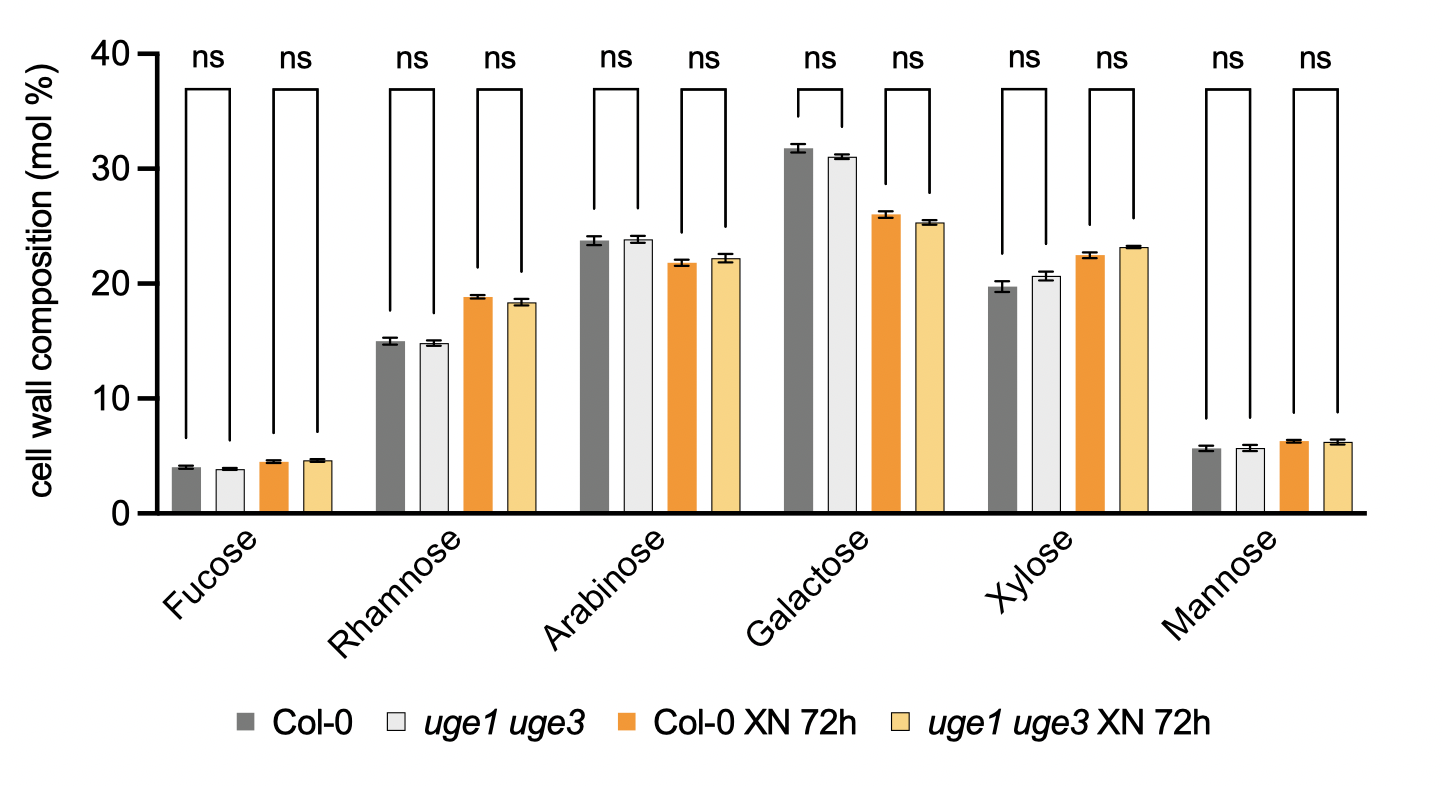


**Figure S5: Cell wall monosaccharide composition in *uge1 uge3* rosettes upon starvation.**

The relative composition of neutral cell wall monosaccharides in whole rosettes of Col-0 and *uge1 uge3* under control conditions (EN) and after 72h XN is depicted as the molar percentage of total cell wall neutral monosaccharide content (mol %). Values are means (n=4) and error bars represent the SEM. A two-way ANOVA and Tukey's multiple comparisons test were performed to investigate differences between Col-0 and *uge1 uge3* (α = 0.05, ns: not significant).


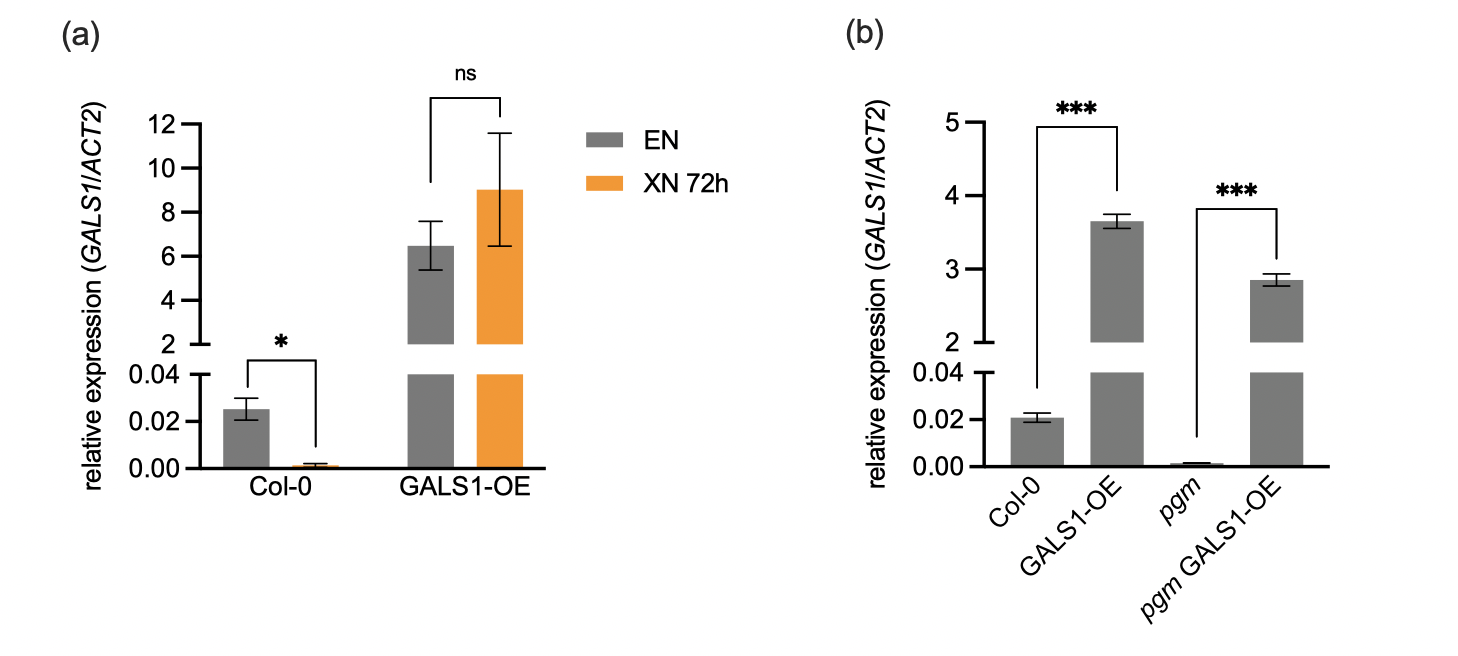


**Figure S6: *GALS1* expression in GALS1-OE lines.**

(a) The relative expression of *GALS1* was determined in rosette leaves of Col-0 and GALS1-OE at the end of the night (EN) and after 72h extended night (XN). Values are means (n=4) and error bars represent the SEM. Asterisks indicate statistically significant differences between XN 24h and EN according to a Student’s t-Test (*p<0.05, ns: not significant). (b) The relative expression of *GALS1* was determined in rosette leaves of Col-0, GALS1-OE, *pgm* and *pgm* GALS1-OE at EN. Values are means (n=3) and error bars represent the SEM. Asterisks indicate statistically significant differences between GALS1-OE and their respective genetic background according to a Student’s t-Test ( ***p<0.001).


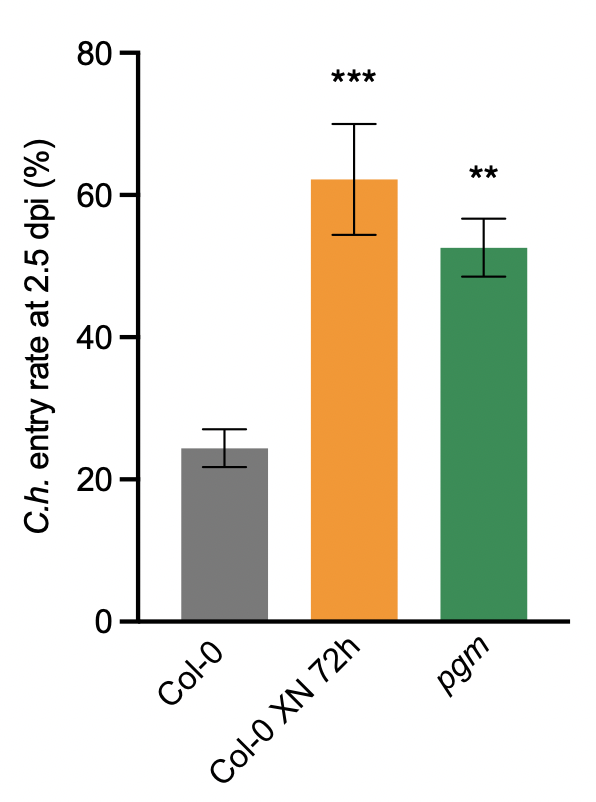


**Figure S7: Dark-induced and periodic starvation lead to increased entry of *Colletotrichum higginsianum*.**

The entry rate of *Colletotrichum higginsianum* at 2.5 days post infection (dpi) was determined microscopically in leaves of Col-0 under control conditions (grey bar), Col-0 after 72h XN (orange bar) and *pgm* under control conditions (green bar). Values are means (n=5) and error bars represent the SEM. Asterisks indicate statistically significant differences to Col-0 according to one-way ANOVA and Dunnett's multiple comparisons test (**p<0.01, ***p<0.001).


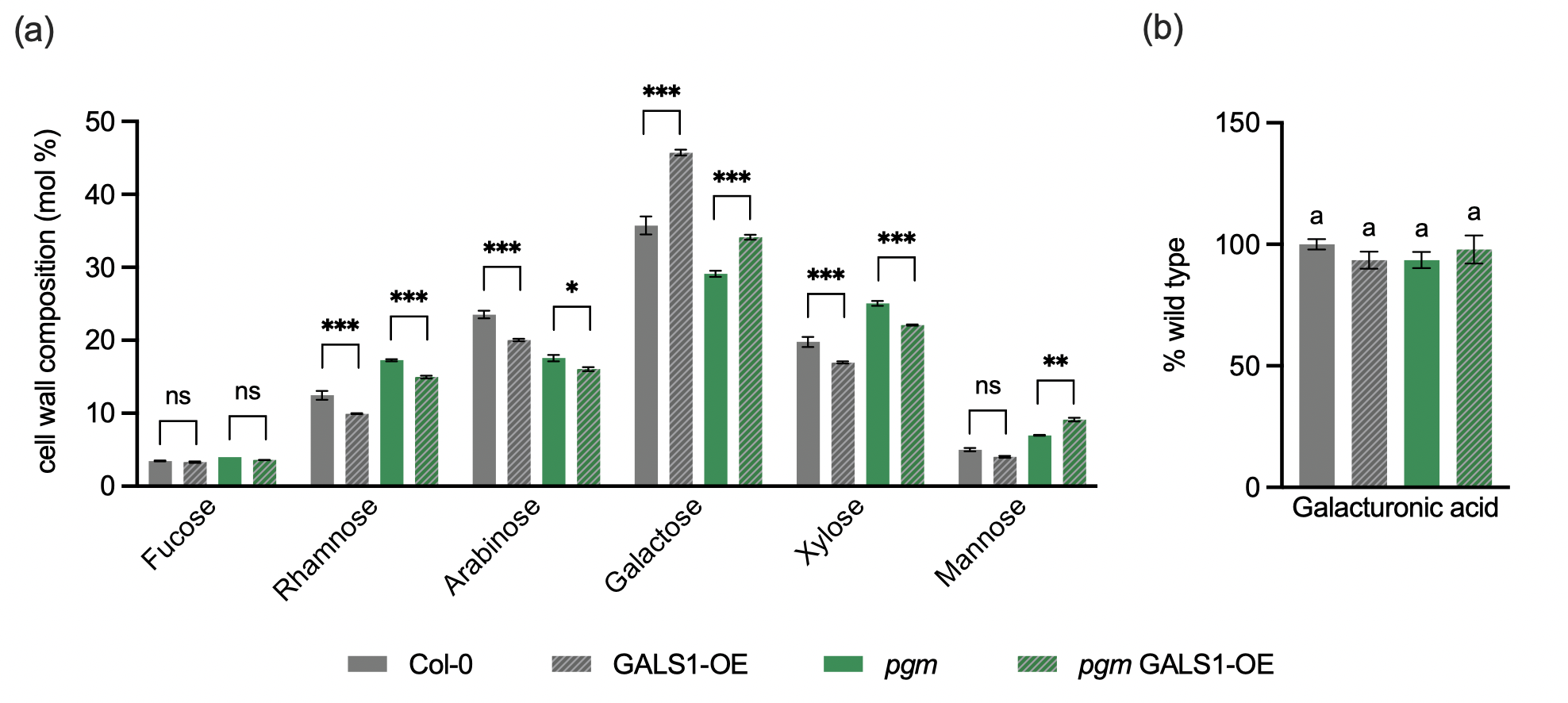


**Figure S8: Cell wall monosaccharide composition in rosettes of GALS1-OE lines.**

(a) The relative composition of neutral cell wall monosaccharides in whole rosettes of Col-0, GALS1-OE, *pgm* and *pgm* GALS1-OE at the end of a 12h night (EN) is depicted as the molar percentage of total cell wall neutral monosaccharide content (mol %). Values are means (n=4) and error bars represent the SEM. Asterisks indicate statistically significant differences between GALS1-OE and their respective genetic background for each monosaccharide according to two-way ANOVA and Tukey's multiple comparisons test (*p<0.05, **p<0.01, ***p<0.001, ns: not significant). The data for galactose mol % from this dataset is also shown in Fig. 4d. (b) Galacturonic acid content of Col-0, GALS1-OE, *pgm* and *pgm* GALS1-OE rosettes at EN was quantified relative to Col-0. Values are means from two independent experiments (n=9) and error bars represent the SEM. Different letters indicate statistically significant differences according to one-way ANOVA and Tukey's multiple comparisons test (α = 0.05).

**Table S1.** **Expression of *BETA-GALACTOSIDASE* (*BGAL*) genes upon periodic and dark-induced starvation.**

Relative gene expression (fold change) of *BGAL* genes in *pgm* rosette leaves compared to Col-0 4, 8 and 12h after start of the night (N) in a 12h/12h (L/D) cycle and in Col-0 rosette leaves after 2, 4, 6, 8, 24 and 48h of extended night (XN) compared to the end of the regular dark phase. Microarray data were taken from Usadel *et al.* (2008). Fold changes larger than 2 are highlighted in bold.

| Gene | AGI | pgm  N 4h | pgm  N 8h | pgm  N 12h | Col-0  XN 2h | Col-0  XN 4h | Col-0  XN 6h | Col-0  XN 8h | Col-0  XN 24h | Col-0  XN 48h |
| --- | --- | --- | --- | --- | --- | --- | --- | --- | --- | --- |
| BGAL1 | AT3G13750 | **5,89** | **5,24** | **2,62** | **2,06** | **2,47** | **2,63** | **2,32** | **2,01** | 1,67 |
| BGAL2 | AT3G52840 | **2,74** | **2,14** | 1,50 | 1,56 | **2,31** | **2,35** | **2,13** | 1,69 | 1,04 |
| BGAL3 | AT4G36360 | 0,54 | 0,30 | 0,20 | 0,99 | 0,65 | 0,45 | 0,33 | 0,18 | 0,07 |
| BGAL4 | AT5G56870 | **13,83** | **24,07** | **21,17** | **5,80** | **22,19** | **37,87** | **31,97** | **22,38** | **24,33** |
| BGAL5 | AT1G45130 | 0,94 | 0,56 | 0,54 | 1,04 | 0,84 | 0,64 | 0,93 | 1,31 | 0,75 |
| BGAL6 | AT5G63800 | 1,71 | 1,64 | 1,39 | 1,19 | **2,03** | 1,81 | **2,07** | **2,13** | **2,31** |
| BGAL7 | AT5G20710 | 0,93 | 0,86 | 1,08 | 0,93 | 0,94 | 0,94 | 0,93 | 1,14 | 1,11 |
| BGAL8 | AT2G28470 | 0,48 | 0,39 | 0,28 | 1,01 | 1,17 | 1,26 | 1,05 | 0,45 | 0,26 |
| BGAL9 | AT2G32810 | 0,83 | 0,76 | 0,68 | 0,86 | 0,84 | 0,87 | 0,67 | 0,54 | 0,37 |
| BGAL10 | AT5G63810 | 0,76 | 0,96 | 0,69 | 0,90 | 1,63 | **4,25** | **8,00** | **4,63** | **2,88** |
| BGAL11 | AT4G35010 | 0,91 | 0,90 | 1,03 | 0,94 | 0,93 | 0,85 | 0,97 | 0,98 | 1,07 |
| BGAL12 | AT4G26140 | 1,24 | 1,39 | 1,34 | 1,68 | 1,59 | 1,83 | 1,72 | 1,71 | 1,55 |
| BGAL13 | AT2G16730 | 0,86 | 0,93 | 0,95 | 0,88 | 0,95 | 0,99 | 1,13 | 1,02 | 0,91 |
| BGAL14 | AT4G38590 | 0,87 | 1,01 | 0,97 | 1,21 | 1,00 | 1,03 | 1,04 | 1,07 | 1,16 |
| BGAL16 | AT1G77410 | 0,73 | 0,85 | 1,04 | 0,83 | 0,84 | 0,90 | 0,95 | 1,01 | 1,10 |
| BGAL17 | AT1G72990 | 0,75 | 0,88 | 1,04 | 1,08 | 0,98 | 0,96 | 1,00 | 0,79 | 1,15 |

**Table S2.** Arabidopsis genotypes used in this study.

| Genotype | AGI | Stock code | Reference |
| --- | --- | --- | --- |
| *bgal1-1* | AT3G13750 | SALK_032664 | Moneo-Sánchez *et al.* (2018) |
| *bgal1-2* | AT3G13750 | SAIL_704_G08 | this work |
| *bgal4-1* | AT5G56870 | SALK_022796 | Moneo-Sánchez *et al.* (2018) |
| *bgal4-2* | AT5G56870 | SALK_093071 | this work |
| *bgal10-1* | AT5G63810 | SAIL_735_F06 | Sampedro *et al.* (2012) |
| *bgal10-2* | AT5G63810 | SALK_039200 | Sampedro *et al.* (2012) |
| 35S::YFP-GALS1 (GALS1-OE) | AT2G33570 |  | Liwanag *et al.* (2012) |
| *gals1-1 gals2-1 gals3-1* (*gals1/2/3*) | AT2G33570,  AT5G44670,  AT4G20170 | Salk_016687,  Salk_ 121802,  WiscDsLox377- 380G11 | Liwanag *et al.* (2012), Ebert *et al.* (2018) |
| *pgm* | AT5G51820 | N210 | Caspar *et al.* (1985) |
| *pgm* GALS1-OE | AT5G51820, AT2G33570 | N210 | this work |
| *pgm uge1-1 uge3-2* | AT5G51820, AT1G12780, AT1G63180 | N210, SALK_019587, SAIL_508_E09 | this work |
| *uge1-1 uge3-2* | AT1G12780, AT1G63180 | SALK_019587, SAIL_508_E09 | Rösti *et al.* (2007) |

**Table S3.** Primers used in this study.

| Name | Method | Sequence | Reference |
| --- | --- | --- | --- |
| BGAL1_q_for | qRT-PCR | GCTTTTTGAATCTCAAGGCGGT |  |
| BGAL1_q_rev |  | AGCTGCCCAGTTGGTGTATG |  |
| BGAL1_q2_for | qRT-PCR  (mutant characterization) | CCGGAACATTCAGAGAGGACA |  |
| BGAL1_q2_rev |  | TTTAGCCACGACCTTGGGAC |  |
| BGAL2_q_for | qRT-PCR | ACTGGACTGGTTGGTTCACG |  |
| BGAL2_q_rev |  | CGGAGAACGCAATGTCTTCG |  |
| BGAL4_q_for | qRT-PCR | TCTGAGTGTTGCAGTGGGTC |  |
| BGAL4_q_rev |  | TGTGACCGGACCAAGAACTC |  |
| BGAL6_q_for | qRT-PCR | TGGTTGGTCTACCGGACTCT |  |
| BGAL6_q_rev |  | TCCCGCCACAGCTAATTTGT |  |
| BGAL10_q_for | qRT-PCR | CTGAGAACTGGCCTGGATGG |  |
| BGAL10_q_rev |  | AACCGAGCAACAGAGTAGGC |  |
| UGE1_q_for | qRT-PCR | TGCCACTGTTTATGGACAACCT |  |
| UGE1_q_rev |  | CTCCATTCCGGTTCTGCCTT |  |
| UGE3_q_for | qRT-PCR | GTGTTGGAAACCCTCGTCGT |  |
| UGE3_q_rev |  | GCAGACGACGAAAACACCAT |  |
| GALS1_q_for | qRT-PCR | TCCAAGGCAATGGGGATTTGA |  |
| GALS1_q_rev |  | TGCGTTCTTCGCTTGGATTG |  |
| ACT2_q_for | qRT-PCR | CTTGCACCAAGCAGCATGAA | Czechowski *et al.* (2005) |
| ACT2_q_rev |  | CCGATCCAGACACTGTACTTCCTT |  |

Reference

Czechowski, T., Stitt, M., Altmann, T., Udvardi, M.K. & Scheible, W.‐R.D. (2005) Genome‐wide identification and testing of superior reference genes for transcript normalization in Arabidopsis. Plant Physiology, 139, 5–17.

Moneo‐Sánchez, M., Izquierdo, L., Martín, I., Hernández‐Nistal, J., Albornos, L., Dopico, B. et al. (2018) Knockout mutants of Arabidopsis thaliana β‐galactosidase. Modifications in the cell wall saccharides and enzymatic activities. Biologia Plantarum, 62, 80–88.
